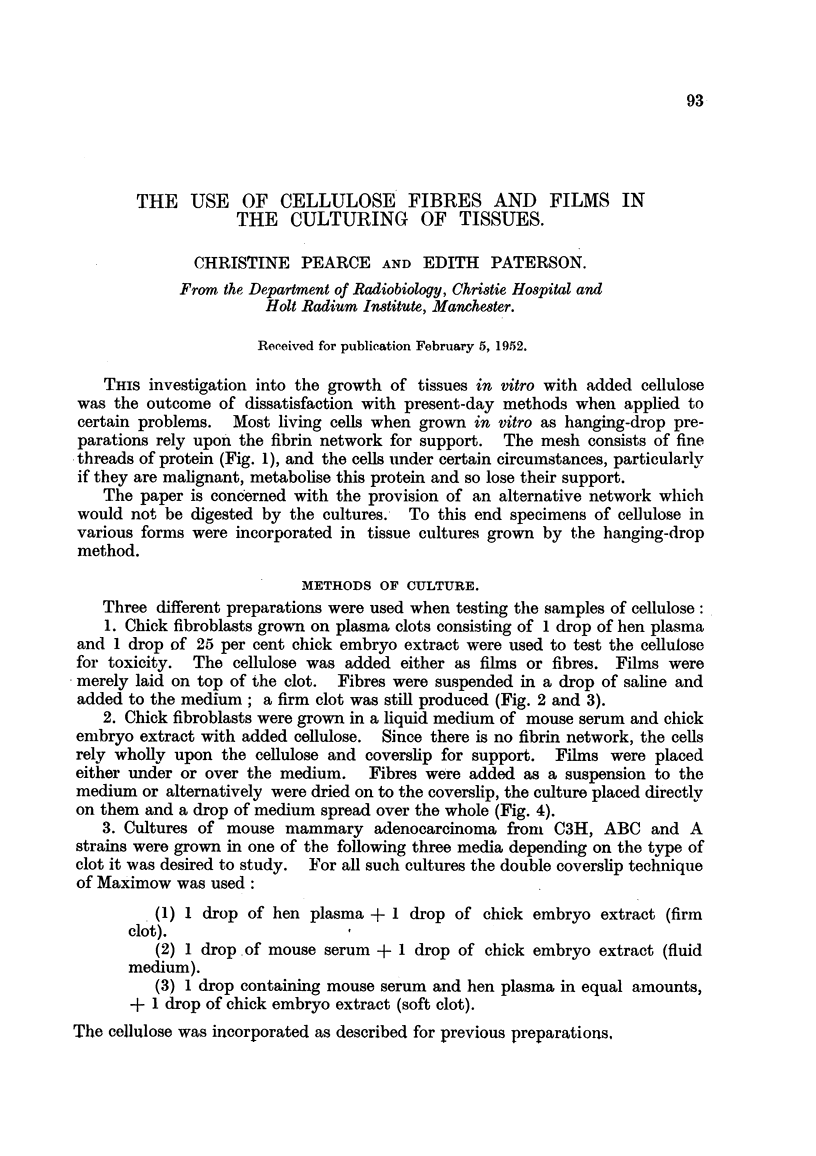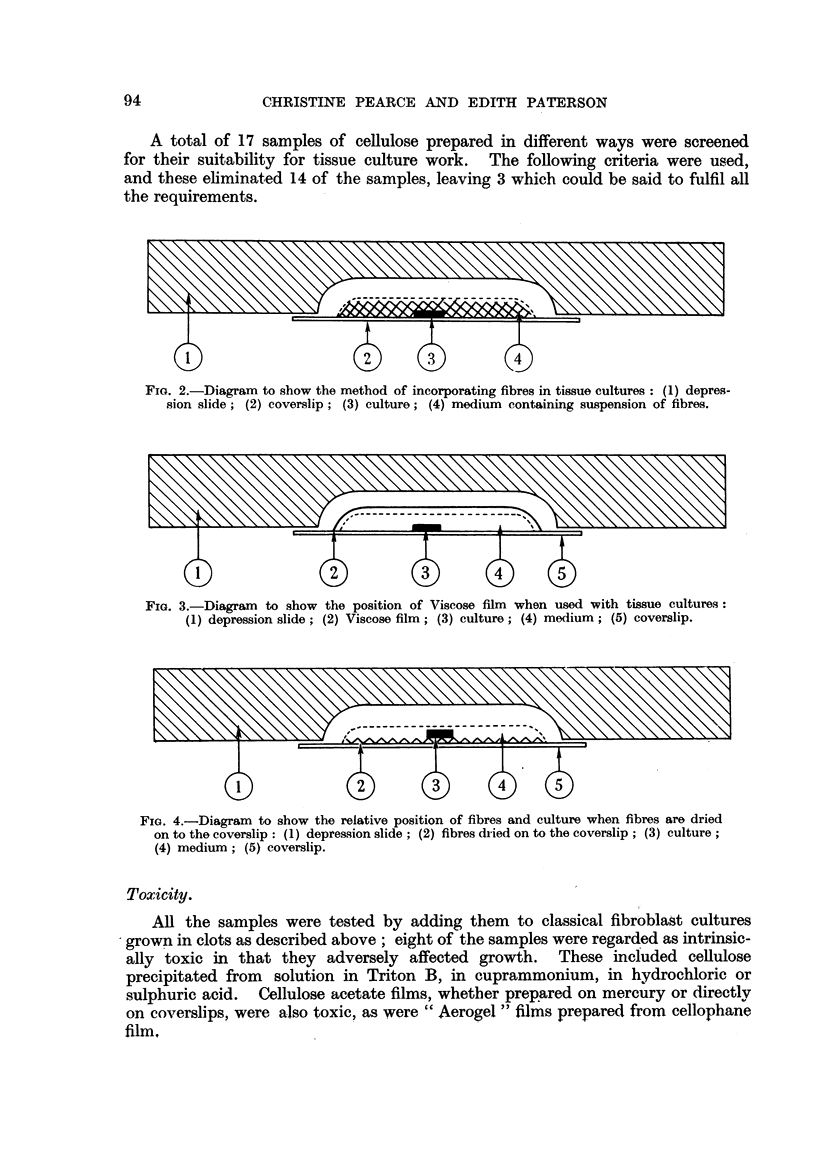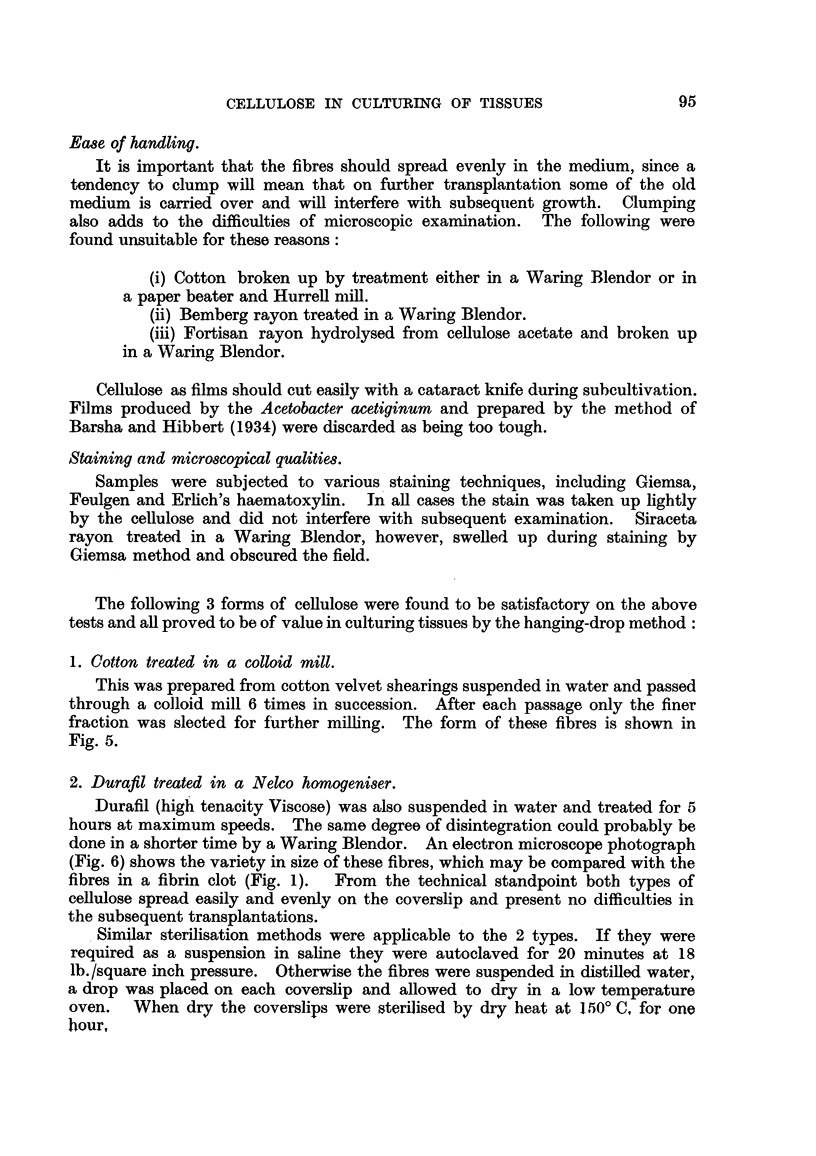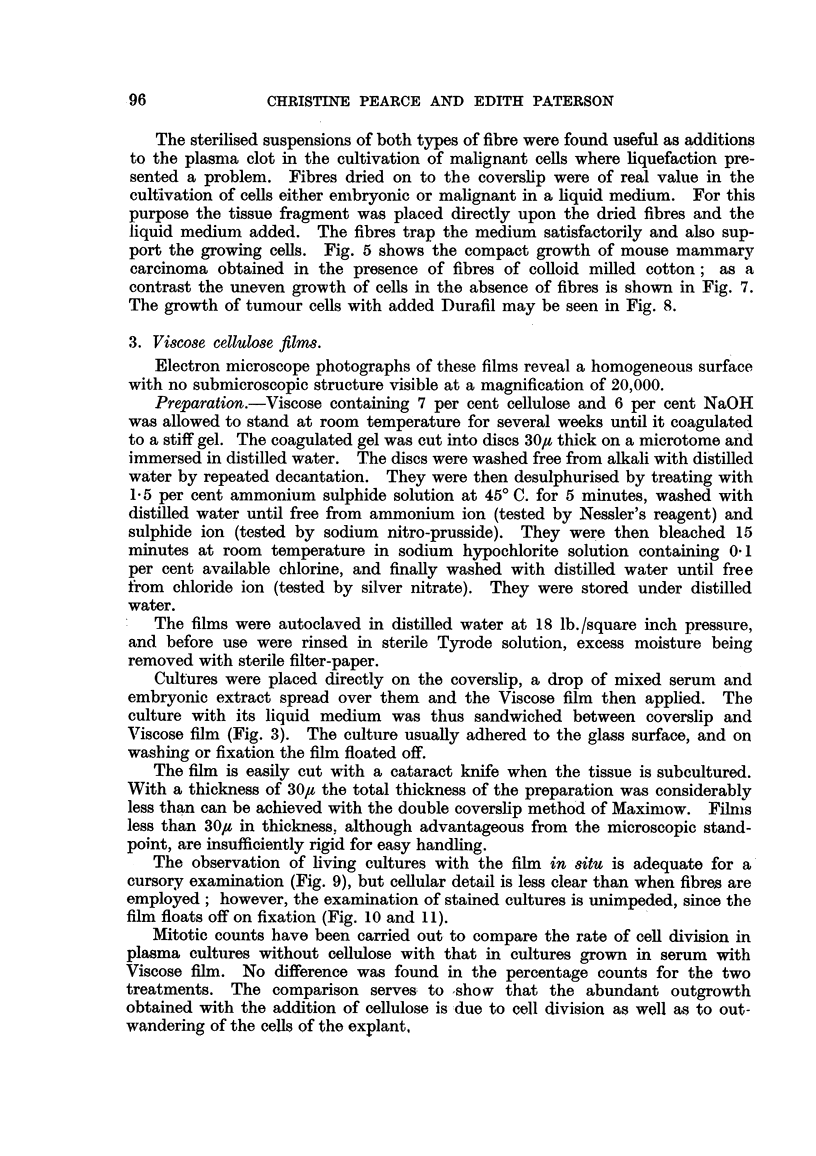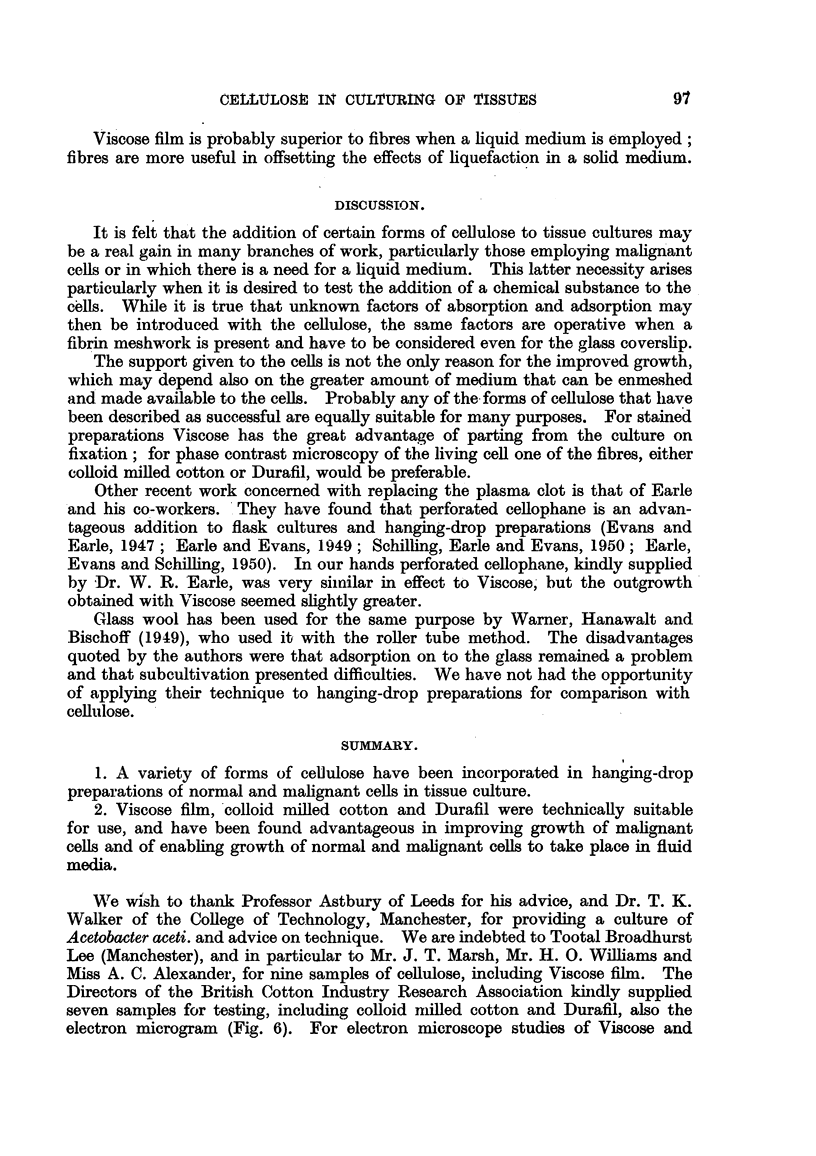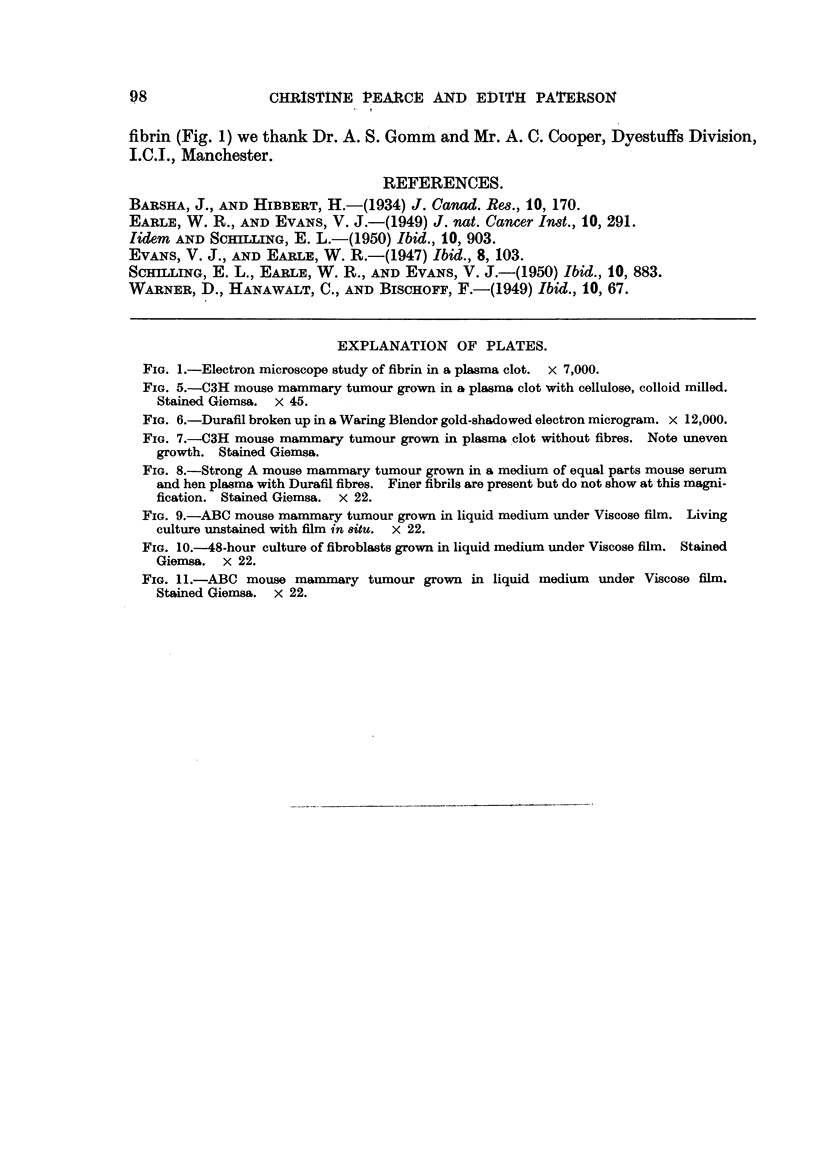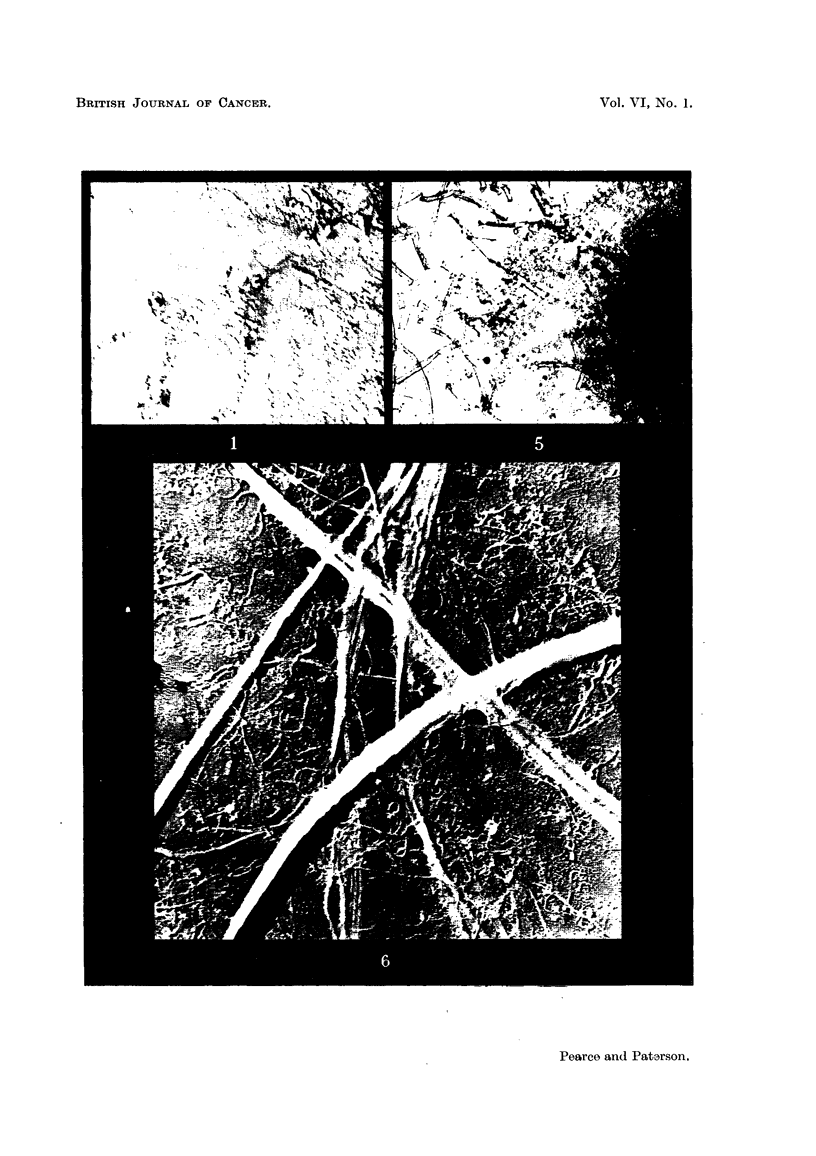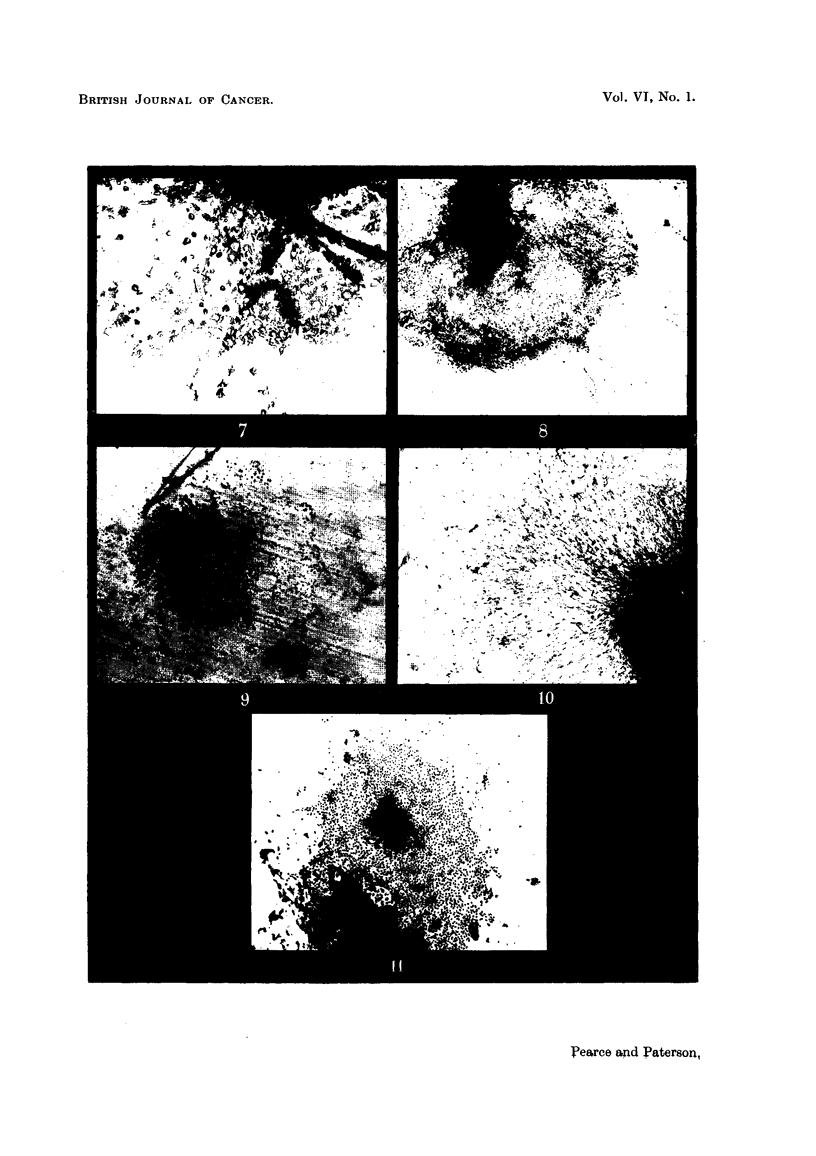# The Use of Cellulose Fibres and Films in the Culturing of Tissues

**DOI:** 10.1038/bjc.1952.10

**Published:** 1952-03

**Authors:** Christine Pearce, Edith Paterson

## Abstract

**Images:**


### THE USE OF CELLULOSE FIBRES AND FILMS IN THE CULTURING OF TISSUES.

CHRISTINE PEARCE AND EDITH PATERSON.

*From the Department of Radiobiology, Christie Hospital and Holt Radium Institute, Manchester.*

Received for publication February 5, 1952.

THIS investigation into the growth of tissues *in vitro* with added cellulose was the outcome of dissatisfaction with present-day methods when applied to certain problems. Most living cells when grown *in vitro* as hanging-drop preparations rely upon the fibrin network for support. The mesh consists of fine threads of protein (Fig. 1), and the cells under certain circumstances, particularly if they are malignant, metabolise this protein and so lose their support.

The paper is concerned with the provision of an alternative network which would not be digested by the cultures. To this end specimens of cellulose in various forms were incorporated in tissue cultures grown by the hanging-drop method.

### METHODS OF CULTURE.

Three different preparations were used when testing the samples of cellulose :

1. Chick fibroblasts grown on plasma clots consisting of 1 drop of hen plasma and 1 drop of 25 per cent chick embryo extract were used to test the cellulose for toxicity. The cellulose was added either as films or fibres. Films were merely laid on top of the clot. Fibres were suspended in a drop of saline and added to the medium ; a firm clot was still produced (Fig. 2 and 3).

2. Chick fibroblasts were grown in a liquid medium of mouse serum and chick embryo extract with added cellulose. Since there is no fibrin network, the cells rely wholly upon the cellulose and coverslip for support. Films were placed either under or over the medium. Fibres were added as a suspension to the medium or alternatively were dried on to the coverslip, the culture placed directly on them and a drop of medium spread over the whole (Fig. 4).

3. Cultures of mouse mammary adenocarcinoma from C3H, ABC and A strains were grown in one of the following three media depending on the type of clot it was desired to study. For all such cultures the double coverslip technique of Maximow was used :

(1) 1 drop of hen plasma + 1 drop of chick embryo extract (firm clot).

(2) 1 drop of mouse serum + 1 drop of chick embryo extract (fluid medium).

(3) 1 drop containing mouse serum and hen plasma in equal amounts, + 1 drop of chick embryo extract (soft clot).

The cellulose was incorporated as described for previous preparations.

A total of 17 samples of cellulose prepared in different ways were screened for their suitability for tissue culture work. The following criteria were used, and these eliminated 14 of the samples, leaving 3 which could be said to fulfil all the requirements.

FIG. 2.—Diagram to show the method of incorporating fibres in tissue cultures : (1) depression slide ; (2) coverslip ; (3) culture ; (4) medium containing suspension of fibres.

FIG. 3.—Diagram to show the position of Viscose film when used with tissue cultures : (1) depression slide ; (2) Viscose film ; (3) culture ; (4) medium ; (5) coverslip.

FIG. 4.—Diagram to show the relative position of fibres and culture when fibres are dried on to the coverslip : (1) depression slide ; (2) fibres dried on to the coverslip ; (3) culture ; (4) medium ; (5) coverslip.

*Toxicity.*

All the samples were tested by adding them to classical fibroblast cultures grown in clots as described above ; eight of the samples were regarded as intrinsically toxic in that they adversely affected growth. These included cellulose precipitated from solution in Triton B, in cuprammonium, in hydrochloric or sulphuric acid. Cellulose acetate films, whether prepared on mercury or directly on coverslips, were also toxic, as were “ Aerogel ” films prepared from cellophane film.

*Ease of handling.*

It is important that the fibres should spread evenly in the medium, since a tendency to clump will mean that on further transplantation some of the old medium is carried over and will interfere with subsequent growth. Clumping also adds to the difficulties of microscopic examination. The following were found unsuitable for these reasons :

(i) Cotton broken up by treatment either in a Waring Blendor or in a paper beater and Hurrell mill.
(ii) Bemberg rayon treated in a Waring Blendor.
(iii) Fortisan rayon hydrolysed from cellulose acetate and broken up in a Waring Blendor.

Cellulose as films should cut easily with a cataract knife during subcultivation. Films produced by the *Acetobacter acetiginum* and prepared by the method of Barsha and Hibbert (1934) were discarded as being too tough.

*Staining and microscopical qualities.*

Samples were subjected to various staining techniques, including Giemsa, Feulgen and Erlich's haematoxylin. In all cases the stain was taken up lightly by the cellulose and did not interfere with subsequent examination. Siraceta rayon treated in a Waring Blendor, however, swelled up during staining by Giemsa method and obscured the field.

The following 3 forms of cellulose were found to be satisfactory on the above tests and all proved to be of value in culturing tissues by the hanging-drop method :

1. *Cotton treated in a colloid mill.*

This was prepared from cotton velvet shearings suspended in water and passed through a colloid mill 6 times in succession. After each passage only the finer fraction was slected for further milling. The form of these fibres is shown in Fig. 5.

2. *Durafil treated in a Nelco homogeniser.*

Durafil (high tenacity Viscose) was also suspended in water and treated for 5 hours at maximum speeds. The same degree of disintegration could probably be done in a shorter time by a Waring Blendor. An electron microscope photograph (Fig. 6) shows the variety in size of these fibres, which may be compared with the fibres in a fibrin clot (Fig. 1). From the technical standpoint both types of cellulose spread easily and evenly on the coverslip and present no difficulties in the subsequent transplantations.

Similar sterilisation methods were applicable to the 2 types. If they were required as a suspension in saline they were autoclaved for 20 minutes at 18 lb./square inch pressure. Otherwise the fibres were suspended in distilled water, a drop was placed on each coverslip and allowed to dry in a low temperature oven. When dry the coverslips were sterilised by dry heat at 150° C. for one hour.

The sterilised suspensions of both types of fibre were found useful as additions to the plasma clot in the cultivation of malignant cells where liquefaction presented a problem. Fibres dried on to the coverslip were of real value in the cultivation of cells either embryonic or malignant in a liquid medium. For this purpose the tissue fragment was placed directly upon the dried fibres and the liquid medium added. The fibres trap the medium satisfactorily and also support the growing cells. Fig. 5 shows the compact growth of mouse mammary carcinoma obtained in the presence of fibres of colloid milled cotton; as a contrast the uneven growth of cells in the absence of fibres is shown in Fig. 7. The growth of tumour cells with added Durafil may be seen in Fig. 8.

3. *Viscose cellulose films.*

Electron microscope photographs of these films reveal a homogeneous surface with no submicroscopic structure visible at a magnification of 20,000.

*Preparation.*—Viscose containing 7 per cent cellulose and 6 per cent NaOH was allowed to stand at room temperature for several weeks until it coagulated to a stiff gel. The coagulated gel was cut into discs 30$\mu$ thick on a microtome and immersed in distilled water. The discs were washed free from alkali with distilled water by repeated decantation. They were then desulphurised by treating with 1·5 per cent ammonium sulphide solution at 45° C. for 5 minutes, washed with distilled water until free from ammonium ion (tested by Nessler's reagent) and sulphide ion (tested by sodium nitro-prusside). They were then bleached 15 minutes at room temperature in sodium hypochlorite solution containing 0·1 per cent available chlorine, and finally washed with distilled water until free from chloride ion (tested by silver nitrate). They were stored under distilled water.

The films were autoclaved in distilled water at 18 lb./square inch pressure, and before use were rinsed in sterile Tyrode solution, excess moisture being removed with sterile filter-paper.

Cultures were placed directly on the coverslip, a drop of mixed serum and embryonic extract spread over them and the Viscose film then applied. The culture with its liquid medium was thus sandwiched between coverslip and Viscose film (Fig. 3). The culture usually adhered to the glass surface, and on washing or fixation the film floated off.

The film is easily cut with a cataract knife when the tissue is subcultured. With a thickness of 30$\mu$ the total thickness of the preparation was considerably less than can be achieved with the double coverslip method of Maximow. Films less than 30$\mu$ in thickness, although advantageous from the microscopic standpoint, are insufficiently rigid for easy handling.

The observation of living cultures with the film *in situ* is adequate for a cursory examination (Fig. 9), but cellular detail is less clear than when fibres are employed; however, the examination of stained cultures is unimpeded, since the film floats off on fixation (Fig. 10 and 11).

Mitotic counts have been carried out to compare the rate of cell division in plasma cultures without cellulose with that in cultures grown in serum with Viscose film. No difference was found in the percentage counts for the two treatments. The comparison serves to show that the abundant outgrowth obtained with the addition of cellulose is due to cell division as well as to outwandering of the cells of the explant.

Viscose film is probably superior to fibres when a liquid medium is employed ; fibres are more useful in offsetting the effects of liquefaction in a solid medium.

### DISCUSSION.

It is felt that the addition of certain forms of cellulose to tissue cultures may be a real gain in many branches of work, particularly those employing malignant cells or in which there is a need for a liquid medium. This latter necessity arises particularly when it is desired to test the addition of a chemical substance to the cells. While it is true that unknown factors of absorption and adsorption may then be introduced with the cellulose, the same factors are operative when a fibrin meshwork is present and have to be considered even for the glass coverslip.

The support given to the cells is not the only reason for the improved growth, which may depend also on the greater amount of medium that can be enmeshed and made available to the cells. Probably any of the forms of cellulose that have been described as successful are equally suitable for many purposes. For stained preparations Viscose has the great advantage of parting from the culture on fixation ; for phase contrast microscopy of the living cell one of the fibres, either colloid milled cotton or Durafil, would be preferable.

Other recent work concerned with replacing the plasma clot is that of Earle and his co-workers. They have found that perforated cellophane is an advantageous addition to flask cultures and hanging-drop preparations (Evans and Earle, 1947 ; Earle and Evans, 1949 ; Schilling, Earle and Evans, 1950 ; Earle, Evans and Schilling, 1950). In our hands perforated cellophane, kindly supplied by Dr. W. R. Earle, was very similar in effect to Viscose, but the outgrowth obtained with Viscose seemed slightly greater.

Glass wool has been used for the same purpose by Warner, Hanawalt and Bischoff (1949), who used it with the roller tube method. The disadvantages quoted by the authors were that adsorption on to the glass remained a problem and that subcultivation presented difficulties. We have not had the opportunity of applying their technique to hanging-drop preparations for comparison with cellulose.

### SUMMARY.

1. A variety of forms of cellulose have been incorporated in hanging-drop preparations of normal and malignant cells in tissue culture.

2. Viscose film, colloid milled cotton and Durafil were technically suitable for use, and have been found advantageous in improving growth of malignant cells and of enabling growth of normal and malignant cells to take place in fluid media.

We wish to thank Professor Astbury of Leeds for his advice, and Dr. T. K. Walker of the College of Technology, Manchester, for providing a culture of *Acetobacter aceti.* and advice on technique. We are indebted to Tootal Broadhurst Lee (Manchester), and in particular to Mr. J. T. Marsh, Mr. H. O. Williams and Miss A. C. Alexander, for nine samples of cellulose, including Viscose film. The Directors of the British Cotton Industry Research Association kindly supplied seven samples for testing, including colloid milled cotton and Durafil, also the electron microgram (Fig. 6). For electron microscope studies of Viscose and fibrin (Fig. 1) we thank Dr. A. S. Gomm and Mr. A. C. Cooper, Dyestuffs Division, I.C.I., Manchester.

### REFERENCES.

BARSHA, J., AND HIBBERT, H.—(1934) *J. Canad. Res.*, **10**, 170.
EARLE, W. R., AND EVANS, V. J.—(1949) *J. nat. Cancer Inst.*, **10**, 291.
*Iidem* AND SCHILLING, E. L.—(1950) *Ibid.*, **10**, 903.
EVANS, V. J., AND EARLE, W. R.—(1947) *Ibid.*, **8**, 103.
SCHILLING, E. L., EARLE, W. R., AND EVANS, V. J.—(1950) *Ibid.*, **10**, 883.
WARNER, D., HANAWALT, C., AND BISCHOFF, F.—(1949) *Ibid.*, **10**, 67.

---

### EXPLANATION OF PLATES.

FIG. 1.—Electron microscope study of fibrin in a plasma clot. × 7,000.

FIG. 5.—C3H mouse mammary tumour grown in a plasma clot with cellulose, colloid milled. Stained Giemsa. × 45.

FIG. 6.—Durafil broken up in a Waring Blendor gold-shadowed electron microgram. × 12,000.

FIG. 7.—C3H mouse mammary tumour grown in plasma clot without fibres. Note uneven growth. Stained Giemsa.

FIG. 8.—Strong A mouse mammary tumour grown in a medium of equal parts mouse serum and hen plasma with Durafil fibres. Finer fibrils are present but do not show at this magnification. Stained Giemsa. × 22.

FIG. 9.—ABC mouse mammary tumour grown in liquid medium under Viscose film. Living culture unstained with film *in situ*. × 22.

FIG. 10.—48-hour culture of fibroblasts grown in liquid medium under Viscose film. Stained Giemsa. × 22.

FIG. 11.—ABC mouse mammary tumour grown in liquid medium under Viscose film. Stained Giemsa. × 22.